# Obesity in spontaneous spondylodiscitis: a relevant risk factor for severe disease courses

**DOI:** 10.1038/s41598-020-79012-8

**Published:** 2020-12-14

**Authors:** Benjamin Schoof, Martin Stangenberg, Klaus Christian Mende, Darius Maximilian Thiesen, Dimitris Ntalos, Marc Dreimann

**Affiliations:** 1 Division of Spine Surgery, Department of Trauma and Orthopaedic Surgery, University Medical Center Hamburg-Eppendorf, Martinistraße 52, 20246 Hamburg, Germany; 2 Department of Neurosurgery, University Medical Center Hamburg-Eppendorf, Martinistraße 52, 20246 Hamburg, Germany

**Keywords:** Risk factors, Signs and symptoms, Comorbidities, Bacterial infection, Outcomes research, Bone

## Abstract

Spondylodiscitis is a serious and potentially life-threatening disease. Obesity is a risk factor for many infections, and its prevalence is increasing worldwide. Thus, the aim of this study was to describe characteristics of obese patients with spondylodiscitis and identify risk factors for a severe disease course in obese patients. Between December 2012 and June 2018, clinical records were screened for patients admitted for spondylodiscitis. The final analysis included 191 adult patients (mean age 64.6 ± 14.8 years). Patient data concerning demographics, comorbidities, surgical treatment, laboratory testing, and microbiological workup were analysed using an electronic database. Patients were grouped according to body mass index (BMI) as BMI ≥ 30 kg/m^2^ or < 30 kg/m^2^. Seventy-seven patients were classified as normal weight (BMI 18.5–24.9 kg/m^2^), 65 as preobese (BMI 25–29.9 kg/m^2^), and 49 as obese (BMI ≥ 30 kg/m^2^). Obese patients were younger, had a higher revision surgery rate, and showed higher rates of abscesses, neurological failure, and postoperative complications. A different bacterial spectrum dominated by staphylococci species was revealed (*p* = 0.019). Obese patients with diabetes mellitus had a significantly higher risk for spondylodiscitis (*p* = 0.002). The mortality rate was similar in both cohorts, as was the spondylodiscitis localisation. Obesity, especially when combined with diabetes mellitus, is associated with a higher proportion of *Staphylococcus aureus* infections and is a risk factor for a severe course of spondylodiscitis, including higher revision rates and sepsis, especially in younger patients.

## Introduction

Spondylodiscitis is a severe and potentially life-threatening disease which continues to be a problem worldwide^1–3^. Patients present with a wide range of clinical symptoms, making diagnosis difficult. Therapy requires prompt and aggressive medical treatment, including proof of germs and surgical therapy, if necessary^3^.

Obesity is a major worldwide health problem that is associated with decreased life expectancy. Furthermore, obesity is known to be a risk factor for surgical site infection and poor outcomes in polytrauma patients^4,5^.

As the prevalence of both spondylodiscitis and obesity are increasing, it is of great interest to identify risk factors and develop treatment paths to improve treatment options and outcomes for patient in this cohort. The aim of this study was to describe characteristics of obese patients with spondylodiscitis and identify risk factors for a severe disease course in obese patients.

### Epidemiology

#### Spondylodiscitis

Pyogenic spondylodiscitis used to be a rare disease, with an incidence of 0.4–2 cases per 100,000 patients each year; however, it has increased in recent decades to 5.8 cases per 100,000 patients in Denmark^1^ and 7.4 cases per 100,000 patients in Japan, which is an increase of 140%^2^. This has been attributed to the higher life expectancy of patients with chronic debilitating diseases^3,8–10^. It is more common in elderly patients, with a peak between 59 and 69 years of age, and there is a male preponderance of 52–69%^6–8^, which is further increased in elderly populations^1,2^. In the current literature, the overall mortality of spondylodiscitis lies between 4.5 and 11%^2,9–11^. The predisposing factors include diabetes mellitus, immunosuppression, intravenous drug use, HIV infection, spinal surgery and dorsal instrumentation, preceding bacteraemia, the presence of intravascular devices, rheumatoid arthritis, and malnutrition^1,2,12–15^. The prevalence of risk factors in large studies (n > 100) vary widely, for example, diabetes mellitus and intravenous drug abuse were found to be 10–37% and 2–79%, respectively^1,2,12,13^. Furthermore, the outcome is significantly worsened when patients present with neurologic compromise, delayed time to diagnosis, or suffer a hospital-acquired infection^9^. Compared to other diseases, spondylodiscitis in obese patients is not well described. Accordingly, although potential risk factors have been identified, their impact remains unclear.

#### Obesity

Obesity is classified according to body mass index (BMI), which is divided into six different categories (see Table 1). Obesity is an increasing worldwide health concern because excess weight gain is associated with an increased risk for several diseases, most notably cardiovascular disease, diabetes, and cancer^16,17^. Obese patients show higher mortality after blunt trauma and have a higher risk of multiple organ failure after severe trauma^4,18^. According to Keaver et al.^19^, the percentage of overweight and obese individuals in England and Ireland by 2030 is proposed to reach 89% and 85% in males and females, respectively, by 2030. By then, obesity is expected to affect 86% of adults in the US^20^. This rise in obesity prevalence has been estimated to lead to an increase in obesity-related coronary heart disease, cancers, and type-2 diabetes mellitus of 97%, 61%, and 21%, respectively^19^. Obesity is associated with decreased life expectancy of approximately 3.3–18.7 years^21,22^. It is known to be a risk factor for surgical site infection in joint arthroplasty and abdominal surgery^5,23^. Additionally, obesity represents an increasing economic burden^24^.

**Table 1:** WHO definition for obesity.

Weight category	BMI (kg/m^2^)	Risk for comorbidities
Underweight	< 18.5	Low
Normal weight	18.5–24.9	Average
Pre-obese	25–29.9	Slightly increased
Obesity I°	30–34.9	Increased
Obesity II°	35–39.9	High
Obesity III°	≥ 40	Very high

#### Pathogens and pathophysiology

In terms of aetiology, spinal infections can be divided into pyogenic, granulomatous (TBC, *Brucella*, fungal), and parasitic infections^8^. Pyogenic and granulomatous spinal infections each make up almost 50% of all spinal infections, whereas parasitic spinal infections are rare^7^. Spondylodiscitis commonly arises from the hematogenous spread of bacteria; the arterial route is more frequent than the venous route^25^.

*Staphylococcus aureus* (29–39%) and streptococci (19%)^7,26^ are the most common microorganisms encountered. The proportion of gram-negative bacteria (mostly *Escherichia coli*) varies between 15 and 39%^7,27–29^.

## Materials and methods

### Data source

The study was conducted at a large university medical center located in central Europe. The hospital is a 1600-bed tertiary care provider hosting in-house departments for medical microbiology and pathology. All methods were conducted in accordance with relevant guidelines and regulations. All experimental protocols were reviewed and approved by the Ethics Committee of the University Medical Center Hamburg-Eppendorf (Hamburg Medical Chamber, Aerztekammer Hamburg; WF-013/20). Informed consent was obtained from all subjects to be included in our prospective database on spondylodiscitis. The evaluation was then carried out as a retrospective analysis of all cases between 2013 and 2018 according to regulations of the Ethics Committee of the University Medical Center Hamburg-Eppendorf.

The manuscript was written following the STROBE guidelines^30^.

### Patient data

The included patients were treated in the division of spine surgery in the department of trauma and orthopaedic surgery. In line with the retrospective study design, clinical records were screened for patients admitted for spondylodiscitis between December 2012 and June 2018.

Patient data concerning demographics, comorbidities, surgical treatment, laboratory testing, and microbiological workup were recorded and analysed using an electronic database. Patients with spondylodiscitis following previous spine surgery were excluded from the study.

### Therapy strategy and approach

We took a multidisciplinary approach involving spine surgeons, microbiologists, and infectious disease specialists. The diagnostic approach included clinical history and physical examination, lab values including C-reactive protein (CRP) levels, white blood cell (WBC) counts, native radiographs, and full spine contrast-enhanced magnetic resonance imaging (MRI) or computer tomography (positron emission tomography [PET-CT]) when MRI was contraindicated. Serial blood cultures were collected from 185 patients (87.3%). Also, transoesophageal echography was performed in 147 patients (69.3%). All patients received intravenous antibiotics followed by an oral course. Anti-infectious treatment was conducted individually for 6 to 12 weeks (mostly intravenous for 14 days followed by an oral course) following the recommendation of our in-house multidisciplinary infections conference. Patients were stratified into a “normal and preobese” group (NWG; BMI of 20–30 kg/m^2^) and an “obese” group (OG; BMI of > 30 kg/m^2^) for risk analysis. Patients with a BMI below 18.5 kg/m^2^ were excluded from the risk analyses due to the low number of patients affected (n = 7). Surgery was performed by four experienced surgeons from the spine surgery team. Surgical treatment, either minimally invasive or conventional/open, depended on the decision of the team. For emergency cases that required surgery during the night shift, the surgeon in charge made the decisions. The patients included were scheduled for surgical treatment due to neurological deficits, septic disease with organ failure or the beginning of organ failure, spinal instability because of major spinal destruction, immobilising back pain, or the failure to respond to adequate conservative treatment including adapted analgesia, physiotherapy, and antibiotic therapy adapted to the spectrum of bacteria. Open surgical treatment was primarily performed, but minimally invasive percutaneous instrumentation was used as an alternative. Radiological guided biopsies were only performed in 20 cases.

### Statistics

Datasets were analysed by Student’s t-test, cross tables, and chi-square tests using IBM SPSS V 25 (IBM, Armonk, NY, USA) and Microsoft Excel (Microsoft).

Odds ratios (OR) were computed for all 2 × 2 crosstabulations with chi-square tests for statistical significance in binary coded variables. Non-parametric data was analysed using Kruskall–Wallis and Mann–Whitney-U test. *P*-values of 0.05 or less were considered significant.

## Results

### Demographics

**Figure 1:**
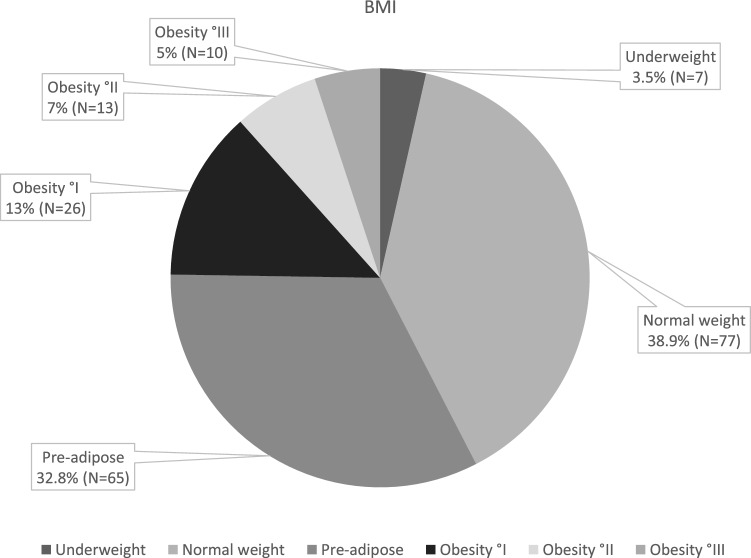
BMI of the 198 patients included to our study.

We identified a total of 212 patients with spondylodiscitis (n = 140, 66% male), aged 64.6 ± 14.8 years. Body weight and body length records were incomplete for 14 patients, so these patients were excluded. Of the remaining 198 patients, 7 were underweight (3.5%), 77 were normal weight (38.9%) and 65 were preobese (32.8%). Among the patients with obesity, 26 were classified as class I (13.1%), 13 as class II (6.6%), and 10 as class III (5.1%), according to the World Health Organization (Fig. 1). The underweight patients were excluded as this represents a disease of its own. Final analysis was performed for the remaining 191 patients.

The mean duration of hospital stay was 27.2 ± 20.7 days, and the mean stay in the intensive care unit (ICU) was 7.3 ± 15.1 days. The mean BMI was 26.9 ± 7.0 kg/m^2^. When comparing the groups, there was no significant difference in the duration of hospital stay (NWG 24.8 ± 15.3; OG 28.6 ± 18.4; *p* = 0.2). The ICU stay was longer for obese patients, but this difference was not significant (NWG 6.6 ± 13.4, OG 7.0 ± 12.1; *p* = 0.2; Table 2).

**Table 2:** Demographics. There is no statistical significance concerning revision surgery, mortality, hospital stay, ICU stay, or affected location but obese patients need to be surgically revised more often than normal weight patients.

Demographics	Normal and preobese		Obese		Significance (*p*)
	BMI < 30		BMI ≥ 30		(student *t *test/Chi^2^ test)
Age (years)	65.8 ± 14.9		63.3 ± 13.8		0.61
Female gender	35.2%		32.7%		0.82
Revision surgery	14.1%		25%		0.08
Mortality	12%		12.2%		0.96
Stay (d)	24.8 ± 15.3		28.6 ± 18.4		0.2
Stay ICU (d)	6.6 ± 13.4		7.0 ± 12.1		0.2
Localization	Percent N		Percent N		
Cervical	17.6%	(25/142)	12.2%	(6/49)	0.77
Thoracic	21.1%	(30/142)	26.5%	(13/49)	
Lumbar	52.8%	(75/142)	53.1%	(26/49)	
Disseminated	8.5%	(12/142)	8.2%	(4/49)	

### Localisation

Spondylodiscitis was localised in the cervical spine in 31 patients (16.2%), in the thoracic spine in 43 patients (22.5%), in the lumbar spine in 101 patients (52.9%), and in a disseminated location in 16 patients (8.4%).

### Clinical findings

The groups did not show any significant differences concerning age, gender distribution, rate of revision surgery, general mortality, patient stay, or localisation (Table 2). Overall, there was a trend towards younger age in patients with diagnosed obesity °I-III compared to patients with normal or pre-obese bodyweight (median 70 vs. 65 years, U = 3084.5, Z = − 1183, *p* = 0.237, Mann–Whitney-U test). In a more detailed analysis of the WHO subgroups using one-way ANOVA and Kruskall-Wallis test, mean age for those groups was: Underweight: 56 years; Normal: 65 years; Preobese: 73 years; Obesity °I: 70 years; Obesity °II: 65 years; Obesity °III 53 years. Kruskall Wallis test showed an effect between patient age and obesity *p* = 0.044, however this was based solely on the comparison between Preobese and Obesity °III patients *p* = 0.05 Bonferroni with Dunn’s correction for multiple tests. All other pairwise comparisons were not statistically significant.

Pre-existing conditions were comparable, except for a significantly higher rate of diabetes mellitus in the OG (10.6% for the NWG vs. 28.6% for the OG; *p* = 0.002). The rates of non-insulin-dependent diabetes mellitus (NIDDM) were similar between the groups. The OR was 3.387 for obese patients with diabetes versus normal weight patients with diabetes. Hepatitis B occurred significantly more often in the NWG (9.9% for the NWG vs. 0.0% for the OG; *p* = 0.022; Table 3). There was no difference in mortality rate between the two groups (12% for the NWG vs. 12.2% for the OG *p* = 0.34). Mortality rates were higher in our tertiary care centre compared to the current literature^8,12,26^.

**Table 3:** Pre-existing medical conditions.

Pre-existing medical conditions	Normal and Preobese	N	Obese	N	Significance (Chi^2^ Sign.)
	BM < 30 (%)		BMI ≥ 30 (%)		
Coronary heart disease	21.1	30/142	18.4	9/49	0.679
Cardiac insufficiency	13.4	19/142	24.5	12/49	0.069
Endocarditis	1.4	2/142	6.1	3/49	0.075
Chronic cystitis	5.6	8/142	4.1	2/49	0.674
Chronic renal failure	16.2	23/142	24.5	12/49	0.196
Dialysis	1.4	2/142	4.1	2/49	0.260
Diabetes mellitus (IDDM + NIDDM)	10.6	15/142	28.6	14/49	0.002
NIDDM	4.9	7/142	4.1	2/49	0.809
Malignoma	23.2	33/142	18.4	9/49	0.478
Osteoporosis	6.3	9/142	2.0	1/49	0.244
Ethanol abuse	9.2	13/142	2.0	1/49	0.099
Intravenous drug abuse	7.0	10/142	0.0	0/49	0.056
Stroke	5.6	8/142	6.1	3/49	0.899
COPD	11.3	16/142	16.3	8/49	0.357
HIV	2.1	3/142	0.0	0/49	0.305
Hepatitis B	9.9	14/142	0.0	0/49	0.022
Liver cirrhosis	4.9	7/142	6.1	3/49	0.747
Rheumatoid disease	6.3	9/142	8.2	4/49	0.662
M. Parkinson	2.8	4/142	0.0	0/49	0.235
Organ transplant	2.1	3/142	4.1	2/49	0.457

Most concomitant diseases appear similarly in both cohorts. Diabetes mellitus (Insulin Dependent Diabetes Mellitus) occurs significantly more often in obese patients, and hepatitis B occurs significantly more often in normal weight people. Cardiac insufficiency and endocarditis occur more often in obese patients (but this is not statistically significant). Ethanol and intravenous drug abuse occur more often in the normal weight group.

There was a slightly higher rate of epidural abscesses in the OG (53.1%) compared to the NWG group (45.1%; *p* = 0.334), as well as psoas abscesses (26.5% for the OG vs. 19.7% for the NWG group; *p* = 0.317).

Neurological deficits affected a higher number of obese patients (26.5%, n = 13), compared to NWG (18%, n = 25), but this result was not statistically significant (*p* = 0.439). In four cases, the loss of function was reversible (one in the OG, three in the NWG) by the time they were discharged from the hospital. Neurological deterioration was only found in two cases in the NWG (none in the OG), most likely due to an individual severe illness course with acute kidney failure followed by sepsis and pneumonia in the second case.

### Laboratory findings

Preoperative CRP counts were significantly higher in the OG (142.3 mg/L) compared to the NWG group (113.3 mg/L; reference range of < 5 mg/L; *p* = 0.038), but there was no significant difference in leukocyte levels between obese patients (10.3 × 10^9^ cells/L) and normal weight patients (11 × 10^9^ cells/L; reference range of 3.8–11 × 10^9^ cells/L; *p* = 0.925).

### Complications

A higher rate of revision surgery was found in the OG (24.5% for the OG [n = 12] vs. 14.1% for the NWG [n = 20]; *p* = 0.09). Obese patients had a three times higher chance of unplanned revision surgery (OR 2.8, RR 2.2) compared to normal weight patients. Also, patients who required unplanned revision surgery had a higher BMI compared to patients who did not (29.8 and 26.5 kg/m^2^, respectively). Regarding in-hospital complications, a significant difference and doubled rate of sepsis (16.2% for the NWG vs. 32.7% for the OG, *p* = 0.01; OR 1.7, RR 1.4) and liver failure (1.4% for the NWG vs. 8.2% for the OG; *p* = 0.02) was found. There were no other statistically significant differences for any other complication, except for a trend towards cardiac decompensation in the OG (7.0% for the NWG vs. 16.3% for the OG; *p* = 0.06; Table 4).

**Table 4:** Complications according to body-weight.

Complications	Normal and Preobese	N	Obese	N	Significance (Chi^2^ test)
	BM < 30 (%)		BMI ≥ 30 (%)		
Revision surgery	14.1	36/142	25.0	12/49	0.08
Renal failure	16.2	23/142	26.5	13/49	0.11
Myocardial infarction	1.4	2/142	0.0	0/49	0.4
Cardiac decompensation	7.0	10/142	16.3	8/49	0.06
Stroke	1.4	2/142	0.0	0/49	0.4
Pneumonia	7.7	11/142	12.2	6/49	0.34
Sepsis	16.2	23/142	32.7	16/49	0.01
Liver failure	1.4	2/142	8.2	4/49	0.02
Shock	7.0	10/142	14.3	7/49	0.13
Delirium	5.6	8/142	6.1	3/49	0.9
Neurological deterioration	1.4	2/142	0.0	0/49	0.4
Thrombosis	0.7	1/142	0.0	0/49	0.56

There are significantly higher rates of sepsis and liver failure in obese patients. Revision surgery and cardiac decompensation rates are almost significant for obese patients.

### Pathogens

**Figure 2:**
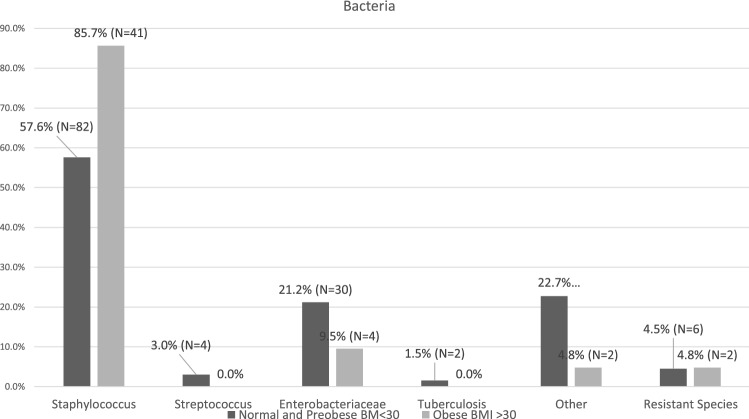
Bacteria that occur in obese and normal weight patients. Known as the most frequent pathogen, staphylococcus species occur substantially more often in obese patients with spondylodiscitis. In some cases, more than one pathogen was isolated.

Bacteria were isolated from blood cultures in 56.4% of OG patients and 47.2% of NWG patients. Intraoperative samples were positive in 65.5% of the NWG and 57.1% of the OG (*p* = 0.54). There was a significant difference concerning the isolated bacterial species responsible for spondylodiscitis between the groups. The NWG group presented significantly fewer staphylococcus species (57.6%) than the OG (85.7%; *p* = 0.019). However, both groups showed the same rates for resistant species (4.5% for the NWG vs. 4.8% for the OG; *p* = 0.236). All other differences were not significant (Fig. 2).

### Treatment

There was no significant difference in the NWG group or the OG in terms of the chosen surgical technique (Table 5). Conservative treatment was carried out in 4.3% of patients in the NWG and 6.1% in the OG. Most patients in the two groups were treated surgically by a posterior approach (40.4% for the NWG vs. 44.9% for the OG; *p* = 0.511) followed by a posterior-anterior approach (27.0% for the NWG vs. 34.7% for the OG; *p* = 0.511).

**Table 5:** Surgical approach.

Surgical approach	Normal and Preobese		Obese		Significance (Chi^2^ test)
	BMI < 30 (%)	N	BMI ≥ 30 (%)	N	
Conservative	4.3	6/142	6.1	3/49	*p* = 0.511
Posterior	40.4	58/142	44.9	22/49	
Anterior	13.5	19/142	8.2	4/49	
Posterior–anterior	27.0	38/142	34.7	17/49	
Anterior–posterior	5.7	8/142	2.0	1/49	
Jamshidi- Biopsy	9.2	13/142	4.1	2/49	

Treatment of patients in the obese group is congruent to treatment of the normal weight group. Even conservative treatment shows equal rates in both rates.

## Discussion

Infectious spondylodiscitis is not an uncommon disease, with an increasing prevalence reported in western society in recent decades^1,2^. The prevalence of obesity has also increased across the world^19,20^. Accordingly, the correlations and effects of spondylodiscitis and obesity on each other are of considerable interest.

### Patient data

Our patient cohort had a mean age of 64.6 years, which is consistent with other studies that identified spondylodiscitis as the main manifestation of hematogenous osteomyelitis in patients older than 50 years of age^31,32^. In the NWG, the average age was 65.8 years, and the OG had an average age of 63.3 years. It is interesting to note that when only patients with obesity grades 2 and 3 were included, classified according to the World Health Organization, the average age was 59.7 years, and when grade 3 alone was considered, the average age was only 54.8 years. These patients still belong to the over 50 age group, but they are noticeably younger than normal weight patients who suffered spondylodiscitis.

### Clinical and laboratory findings

Preoperative CRP counts were significantly higher in the OG compared to the NWG group. Unexpectedly, although not significant, obese patients showed lower leukocyte levels than normal weight patients, which is in contrast to common findings concerning the impact of obesity on immunity. Thus, the underlying reason remains unclear^33^.

In our cohort, obese patients showed a higher rate of epidural abscess (53.1%) compared to the NWG group (45.1%), as well as psoas abscess (26.5% and 19.7%, respectively). Consequently, obese patients had a higher rate of neurological dysfunction (26.5%, n = 13), whereas only 18% of the NWG showed neurological dysfunction. In four cases, the loss of function was reversible (one in the OG, three in the NWG) by the time of discharge from the hospital. The rates of the NWG were comparable to former studies^9,26,34^, but the rates in the OG were noticeably higher.

Additionally, we found a trend toward a higher rate of revision surgery in the OG (OG 25% vs. NWG 14.1%; *p* = 0.08; OR 2.8, RR 2.2). Also, patients who underwent unplanned revision surgery had a higher BMI compared to those who did not (29.8 and 26.5 kg/m^2^, respectively), which is similar to the results observed for arthroplasty surgery^35^.

In our study, most patients with pyogenic spondylodiscitis suffered from concomitant diseases. The most common and significant in our study were diabetes mellitus (28.6%) in the OG and hepatitis B (9.9%) in the NWG. In accordance with former studies, diabetes mellitus, renal insufficiency, and cardiac diseases are the most frequent concomitant diseases in patients with spondylodiscitis^36^. In our cohort, we found an equal occurrence of NIDDM in the NWG and OG groups, and there was no significant difference for renal insufficiency (NWG 16.2%, OG 24.5%; *p* = 0.196; OR 1.687). In our data, we found a trend but no significant difference in cardiac disease prevalence (NWG 13.4%, OG 24.5%; *p* = 0.069; OR 2.1). When compared to data from a primary care study addressing the subject of obesity and diabetes, the present collective shows comparable results. Bramlage et al. reported an OR of 1.67 (1.54–1.81) for the overweight population (BMI 25– < 30) and an OR of 3.52 (3.23–3.83) for the obese population (BMI > 30), which are similar to the results of the present study, with an OR of 3.387 (1.493–7.680, *p* = 0.002) for obese patients^37^.

Mortality rates were higher in our tertiary care centre compared to the current literature due to a higher than average number of patients living with heavy pre-existing diseases in our center (ASA score > 3)^8,12,26^. Another explanation might be related to the high proportion of inpatient transfers from other spine centers (99/191 = 52%) where no care was possible due to the severity of the disease or where conservative therapy had failed.

### Pathogens

With regard to pathogens isolated from blood cultures, 56.4% of patients in the OG and 47.2% in the NWG showed positive samples. Perioperative samples were positive in 65.5% of the NWG and 57.1% of the OG. These results are comparable to former studies^15,36^.

Significantly different bacteria were isolated in both groups. Previous studies demonstrated that staphylococci species are the main pathogens responsible for spondylodiscitis, followed by streptococci and Enterobacteriaceae^32,36,38^. In the OG, staphylococci represented almost 86% (57.6% of the NWG) of all infections, followed by Enterobacteriaceae (9.5%). This greatly outperforms rates reported in the current literature^7,26^. We do not know the underlying reason for the high incidence of staphylococcus species that caused spondylodiscitis in obese patients. Coincidentally, *Pseudomonas aeruginosa*, an uncommon cause of pyogenic spondylodiscitis, was isolated in 48% of 61 patients who reported intravenous drug use^39^. Most likely, obesity together with recurring lesions in skin folds creates a favourable environment for staphylococci infection as intravenous drug use does for *Pseudomonas aeruginosa*.

### Complications

Significant rates of in-hospital complications were found for liver failure and sepsis in obese patients with spondylodiscitis.

### Localisation

No difference concerning localisation was seen between the obese and normal weight cohort, with a similar distribution for lumbar, thoracic, and cervical spondylodiscitis (Table 2) compared to current data^40^.

### Surgical treatment

Bodyweight had no impact on the surgical approach chosen for treatment; however, it is commonly understood that surgery on obese patients is highly challenging. It is of great importance that the ideal therapy is not denied to the patient due to obesity. To our knowledge, there are no further data regarding whether obesity is a limiting factor in spinal surgery.

### Limitations

The limitations of this study include its single-center retrospective study design and that a relatively high number of patients (n = 21/212; 10%) were excluded due to a lack of information in our electronic database or an underweight body mass index.

## Conclusion

Obese patients have significantly higher rates of postoperative liver failure and sepsis compared to normal weight patients. There were higher rates of revision surgery and cardiac decompensation in the obese group, but they were not statistically significant.

Abscesses occurred more often in the obese group, but also not statistically significant. Obese patients are younger than normal weight patients when they suffer from spondylodiscitis. Obese patients suffering from diabetes represent a special risk profile for spondylodiscitis. Obese patients demonstrate a unique bacterial spectrum compared to normal weight patients.

Obese patients do not have a higher mortality rate than normal weight patients with spondylodiscitis. Summarised, obese patients show a more severe course of spondylodiscitis, but we were unable to completely substantiate these presumptions as the length of hospital stay and mortality rate were similar between the two groups. As both obesity and spondylodiscitis have increased over the past couple of decades, it is necessary to focus more attention on this patient cohort.
